# An Insight into the Role of Non-Porphyrinoid Photosensitizers for Skin Wound Healing

**DOI:** 10.3390/ijms22010234

**Published:** 2020-12-28

**Authors:** Mariana C. S. Vallejo, Nuno M. M. Moura, Maria Amparo Ferreira Faustino, Adelaide Almeida, Idalina Gonçalves, Vanda V. Serra, Maria Graça P. M. S. Neves

**Affiliations:** 1 LAQV-REQUIMTE, Department of Chemistry, University of Aveiro, 3810-193 Aveiro, Portugal; mariana.vallejo@ua.pt (M.C.S.V.); faustino@ua.pt (M.A.F.F.); 2 CESAM, Department of Biology, University of Aveiro, 3810-193 Aveiro, Portugal; aalmeida@ua.pt; 3 CICECO, Department of Materials and Ceramic Engineering, University of Aveiro, 3810-193 Aveiro, Portugal; idalina@ua.pt; 4 Centro de Química Estrutural, Instituto Superior Técnico, Universidade de Lisboa, Av. Rovisco Pais 1, 1049-001 Lisboa, Portugal; vanda.serra@tecnico.ulisboa.pt

**Keywords:** wound healing, photosensitizer, photodynamic process, reactive oxygen species, skin, photobonding

## Abstract

The concept behind photodynamic therapy (PDT) is being successfully applied in different biomedical contexts such as cancer diseases, inactivation of microorganisms and, more recently, to improve wound healing and tissue regeneration. The effectiveness of PDT in skin treatments is associated with the role of reactive oxygen species (ROS) produced by a photosensitizer (PS), which acts as a “double agent”. The release of ROS must be high enough to prevent microbial growth and, simultaneously, to accelerate the immune system response by recruiting important regenerative agents to the wound site. The growing interest in this subject is reflected by the increasing number of studies concerning the optimization of relevant experimental parameters for wound healing via PDT, namely, light features, the structure and concentration of the PS, and the wound type and location. Considering the importance of developing PSs with suitable features for this emergent topic concerning skin wound healing, in this review, a special focus on the achievements attained for each PS class, namely, of the non-porphyrinoid type, is given.

## 1. Introduction

**Figure 1:**
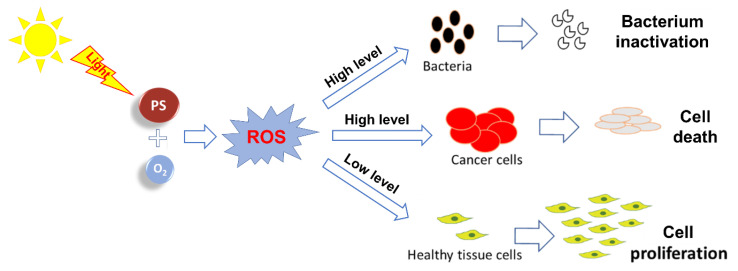
Proposed mode of action of photosensitizer (PS)-derived reactive oxygen species (ROS) for different applications.

Photodynamic therapy (PDT) is a versatile therapeutic technique that has been successfully applied throughout the years in cancer treatment [1,2,3,4,5], aged related macular degeneration [3,6] and in the inactivation of microorganisms (antimicrobial PDT = aPDT) under clinical and non-clinical contexts [7,8,9,10]. More recently, the possibility of exploring the approach for improving wound healing/tissue regeneration after a traumatic lesion is attracting the attention of several research groups [11,12,13,14,15]. The localized response to PDT treatment varies according to the conditions in which this therapeutic approach is applied, where a photosensitizer (PS) molecule/drug with the ability to absorb visible light is placed in direct contact with the target tissue. In the presence of dioxygen (^3^O_2_), the PS irradiation, at an adequate wavelength, gives rise to reactive oxygen species (ROS). Depending on the light parameters and on the PS structure, different levels of ROS can be produced [16]. Such ROS levels can be toxic enough to promote in situ (1) cancerous cell destruction [16,17,18] or (2) pathogenic microorganism inactivation, namely, of multidrug-resistant strains [12,19,20,21,22], or in a level, able to induce cell proliferation and tissue regeneration (Figure 1) [11,13,14].

**Figure 2:**
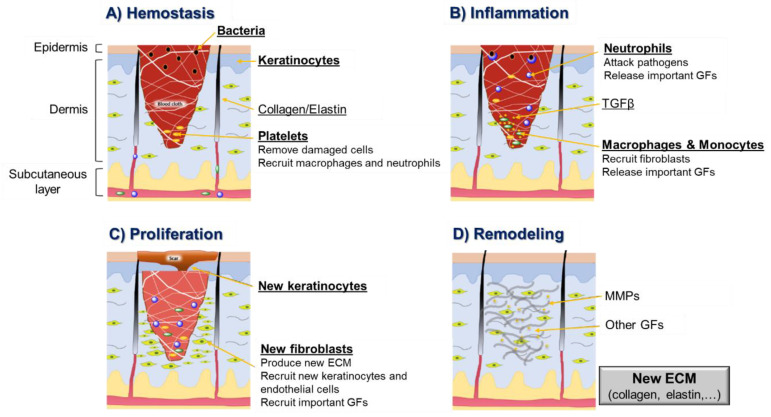
Schematic representation of wound healing stages, highlighting the key molecular and cellular events in each stage (GF—growth factor; TGF—transforming growth factor; MMP—matrix metalloproteinase; ECM—new extracellular matrix).

Although the use of PDT for cell proliferation/tissue regeneration is an encouraging application topic, there is some controversy about its potentiality and mechanism of action involved [23,24,25]. Some studies suggest that the efficiency of this PDT healing treatment is a result of combining the eradication of local infections (that prevent the regenerative process) mediated by ROS and the photobiostimulation of localized cell growth. The use of PDT treatments with low-level energy is gaining special interest for skin wound healing [14]. This promising treatment is particularly relevant since the skin is the largest organ of the human body and is responsible for protecting internal tissues, acting as a physical, chemical and biological barrier against different external agents. When its integrity is compromised by burns, physical trauma or surgery, for instance, it will result in a wound formation that can reach, in certain circumstances, a severe level of gravity for the patient’s health [26]. In some cases, when skin suffers a light trauma, it has the ability for self-regeneration through a sequential four-stage process: (1) hemostasis (the formation of a blood clot), (2) inflammation (the recruitment of inflammatory cells such as macrophages and neutrophils), (3) cell proliferation (the in situ multiplication of fibroblasts, keratinocytes and endothelial cells, and collagen production) and (4) remodeling (the formation and organization of new extracellular matrix = ECM) (Figure 2) [27].

External factors related to the patient (e.g., oxygenation, age, stress, diabetes, obesity, medications, alcoholism, smoking and nutrition) [28,29] can interfere with one or more of the healing phases, compromising the regenerative process. In general, it is completed after 14 days, from the hemostasis phase to the remodeling phase [30]. Nonetheless, the major concern is related to infections created in an open wound, where the rupture of different skin layers allows microbial penetration and consequent proliferation [31]. This barrier rupture can slow down and compromise the regenerative process, creating a high risk for the patient’s life [13].

**Figure 3:**
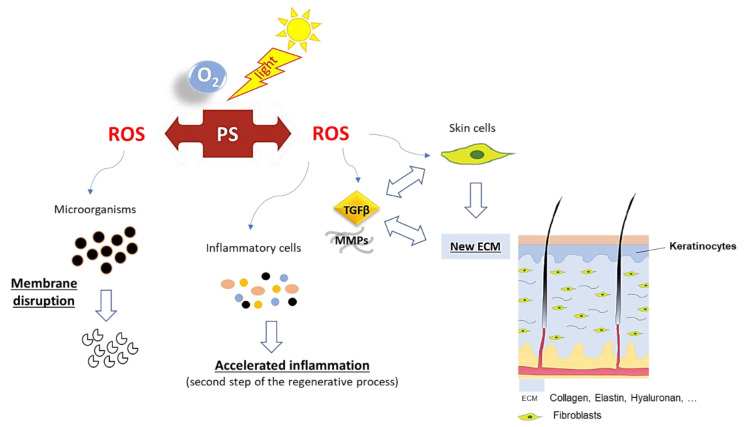
Proposed action pathway for photodynamic therapy (PDT) in skin wound healing.

The possibility of PDT acting as a “double agent” can be of high relevance for skin wound healing. Ideally, ROS produced by activated PS can promote the selective death of microorganisms by acting at different targets such as membrane phospholipids, proteins and nucleic acids [13,32,33], also affecting the expression of virulence factors (e.g., enterotoxins, lipases, thermonucleases and coagulase production) [13,34]. At the same time, PDT can act at the early stage of the regenerative process through the acceleration of the immune system response. More specifically, PDT induces the recruitment of important agents to the wound site, such as neutrophils, myeloid cells, monocytes or macrophages, which promote the elimination of damaged tissue in the wound and the induction of pro-inflammatory cytokines [11,13,14,35]. Additionally, a low amount of ROS not only enhances mitochondrial activity, the respiratory chain and ATP synthesis [36,37] but also recruits important metalloproteinases (MMPs)/growth factors (GFs) and activates signal-regulated kinases [38,39]. All these components are critical for the differentiation and proliferation of skin cells (such as fibroblasts) and extracellular matrix components (such as elastin and collagen) that consequently accelerate the regenerative process [14,38,39,40] (Figure 3). An additional advantage associated with PDT use is related to the fact that ROS action is non-specific, meaning that resistance to the treatment is not likely to be developed by the bacteria [19].

**Figure 4:**
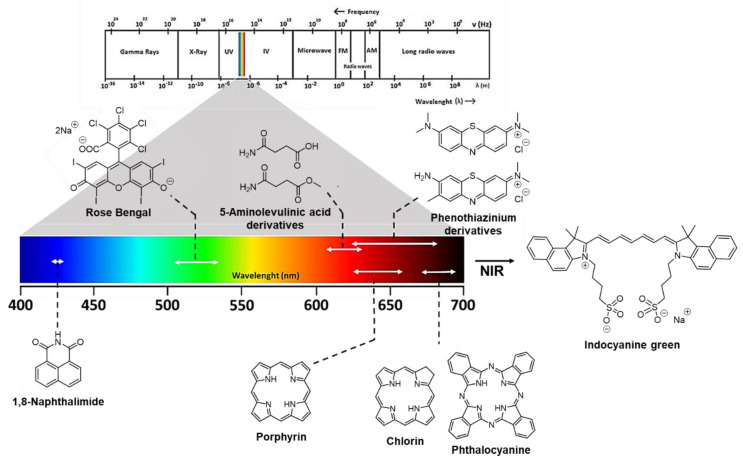
Framework of different PSs used in skin wound healing and their usual activation wavelengths.

PS’ role is particularly relevant for ensuring PDT effectiveness in skin wound healing. Different classes of PS molecules such as phenothiazinium derivatives (e.g., methylene blue and toluidine blue O), xanthene dyes (Rose Bengal) and tetrapyrrolic macrocycles and analogs such as porphyrins, chlorins and phthalocyanines have been studied (Figure 4). Considering that each PS has a characteristic light absorption peak (Figure 4), its selection or design is a key factor and will determine the light wavelength to be used during PDT for producing the required ROS that will inactivate microorganisms and help to induce proliferation without harming the skin cells [13,14,31]. Moreover, the activation of some PS such as Rose Bengal (RB) when in contact with collagen wounds can also promote covalent crosslinks with the consequent attachment of the tissues [41,42].

Most of the studies report light wavelengths applied for wound healing in the “phototherapeutic window” (600–800 nm range), where the tissue light penetration is deeper [11,13], although some PSs also absorb at lower wavelengths (e.g., naphthalimide and Rose Bengal dyes) (Figure 4). It is important to mention that the efficiency of PS excitation/ROS generation and the effects produced are strongly dependent on the light wavelength and on the light parameters (irradiance, irradiation time, light regime, etc.) selected [11,14]. The relevance of and interest in exploring PDT in wound/excision healing are obvious when the numbers of publications and excellent reviews recently published concerning important aspects such as the light source, treatment protocol and wound location are evaluated [11,13,14].

Considering the importance of developing PSs with suitable features for skin wound healing, in this review, the achievements attained using different PS classes are highlighted. The high number of studies concerning the different types of PS prompted us to restrict the discussion to studies obtained with non-porphyrinoid PSs such as phenothiazinium dyes, xanthene dyes (Rose Bengal), phthalimide derivatives, indocyanine green and other photocatalytic systems based on hypericin, fullerene, graphene oxide and inorganic salts/nanoparticles (Ag_3_PO_4_, CuS or TiO_2_). The results obtained with synthetic and natural tetrapyrrolic macrocycles such as porphyrins, chlorins and phthalocyanines will be the subject of another analysis. Thus, in the following sections, the results will be discussed according with the type of photosensitizer.

## 2. Photosensitizing Dyes

### 2.1. Phenothiazinium Derivatives

**Figure 5:**
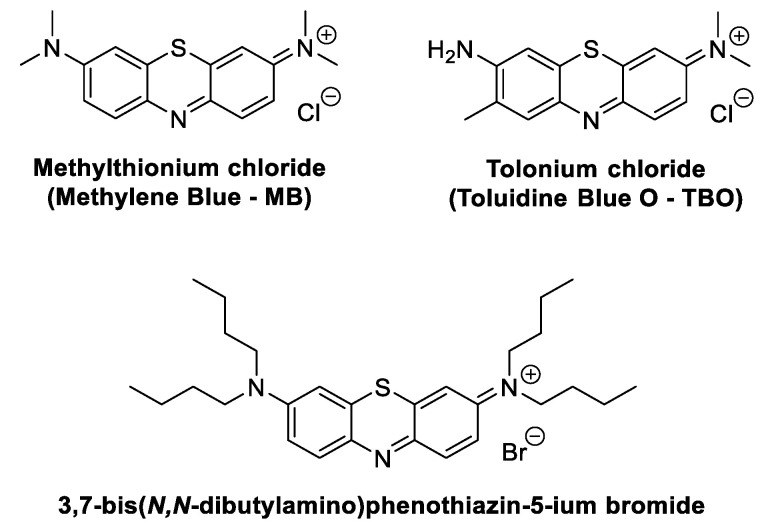
Structures of phenothiazinium derivatives discussed in this review as wound healing agents.

Phenothiazinium derivatives (Figure 5) are a class of synthetic dyes synthesized for the first time in the 19th century. In these derivatives, sulfur and nitrogen atoms are located at positions 9 and 10, respectively, of the phenothiazine-fused tricyclic system central ring [43]. Due to their photochemical and photophysical properties, namely, light absorption above 600 nm, phenothiazinium derivatives have been extensively studied as PSs to be used as anticancer and antimicrobial agents, as well as redox and photochromic materials [44,45,46].

#### 2.1.1. Methylene Blue

A popular PS among the phenothiazinium derivatives that is attracting the attention of different research groups working in the skin wound healing context is methylthioninium chloride, usually known as methylene blue (MB) (Figure 5).

In 2010, Sperandio and coworkers, aiming to clarify some contradictory results concerning the healing effect of PDT in wounds [25,37,47,48,49], reported an interesting study where that effect mediated by methylene blue (MB) was evaluated in wound lesions generated on rats’ dorsa [24]. The study involved 100 rats, which were adequately divided into the following groups: “Control”, where animals with no local or systemic treatment were included; “Dye”, where the animal’s wounds were treated with MB (0.01%) only for 5 min, filling all the excised region; “Laser”, where the wounds were only subject to light irradiation (a diode laser at 660 nm, an input power of 100 mW and a laser beam at 0.028 cm^2^); and finally, “PDT”, where the animal’s wounds were subject to PDT treatment—the same light irradiation protocol as used in the “Laser” group but in the presence of MB. All the irradiations were performed in contact mode for 33 s with a total light dose of 117.85 J/cm^2^. The results revealed, after an evaluation of wound contraction and histologic analyses in predetermined periods, that all the wounds were completely repaired by Day 14. However, the group “Laser” (only irradiation) showed the best results for a less-intense inflammatory infiltrate and smallest wound area after 14 days of follow-up, which was closely followed by the remaining groups (“Control”, “Dye” and “PDT”). Nonetheless, both the “Laser” and “PDT” groups showed a better collagen net organization and faster tissue remodeling, compared to the others. Thus, although the “Laser” group appeared to present a better achievement concerning wound healing, the full advantage of PDT treatment due to its antimicrobial action could not be assessed since the study did not involve infected wounds.

**Figure 6:**
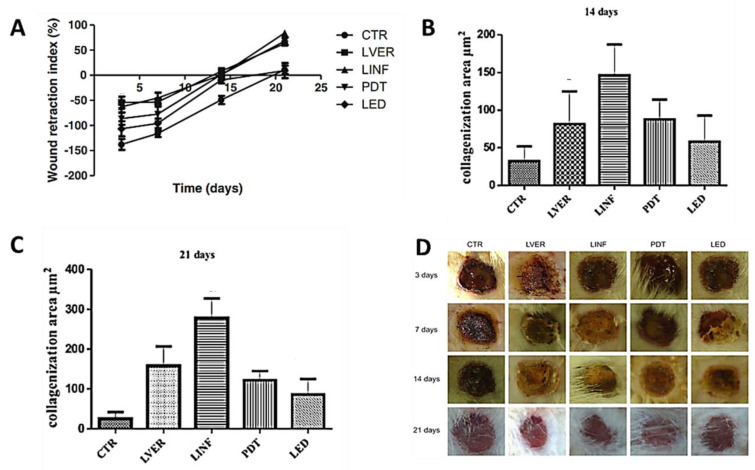
(**A**) Wound retraction index in third-degree burns treated with different light sources; collagenization areas formed at (**B**) 14 days and (**C**) 21 days; (**D**) photographs of skin burns of rats according to the treatment group and sacrifice times (figure adapted from [36] with permission of Springer Nature).

Considering that wound healing can be promoted by PDT treatment but also by using red, green or infrared lights, Catão and his group compared the effects of these therapies in the healing process for third-degree burns induced in rats [36]. The PDT treatment was mediated by MB at a non-cytotoxic concentration (0.5 μg/mL, applied 5 min before irradiation). The study involved one hundred rats, which were divided into five groups including the control group (CTR). The animals treated with red laser light at 660 nm (LVER), infrared light at 780 nm (LINF), and red laser light at 660 nm but in the presence of MB (PDT) received a total daily dose of 40 J/cm^2^ for 21 days; the rats with burns treated with a green light-emitting diode at 520 to 550 nm (LED) received a total light dose of 240 J/cm^2^ per day. The healing process for the burns was monitored for 21 days, and the results, when compared with the animals with burns, without any treatment (CTR), were promising. All the groups, compared to the control group, showed higher wound retraction and a higher capacity to stimulate fibroblasts in the production and maturation of collagen, especially in the first 15 days. However, the highest wound retraction was achieved with the animals treated with infrared light (780 nm) followed by the ones treated with green light and then PDT (Figure 6A). The assessment of the collagenation areas after 14 days (Figure 6B) and 21 days (Figure 6C) also revealed that the best results were achieved by LINF. In this study, the full advantage of the PDT treatment could not be inferred. In fact, Figure 6D shows that there was no apparent improvement of the wounds after the PDT treatment using this dye. As before, the study did not involve infected wounds, and the PS antimicrobial action towards wound bacteria, which could delay the healing process, was not evaluated.

Knowing that PDT therapy mediated by MB is a promising antimicrobial modality [50,51,52], and skin wound healing can be heavily compromised by wound localization, Deyhimi et al. [23] investigated the effects of this procedure in experimental oral mucosal wounds in 48 rat models. In the PDT group, after MB (2%) application for 5 min, the wounds were irradiated with laser light (a diode laser at 660 nm and input power of 25 mW) for 10 s with total light dose of 1.0 J/cm^2^. The treatments were performed between 1–4 and 6–9 days, and the wounds were monitored after 2, 7 and 14 days. A group of animals that did not receive treatment (Control group) and animals that only received the light irradiation (Light group) were used for comparison. The histological results allowed us to conclude that PDT accelerates wound healing in the first 7 days, but the same pattern was not observed in the following days. Moreover, a higher degree of healing was observed in the Control group. Thus, it was established that the light parameters used in this study resulted in a delay of the healing process mediated by PDT and by the light treatments. It was suggested that the inhibitory effect was probably due to the occurrence of light absorption by fibroblast cells. However, the innocuous effect of PDT on the wound healing process during the first days can be important to suppress an infection after an injury due to its antimicrobial effect.

**Figure 7:**
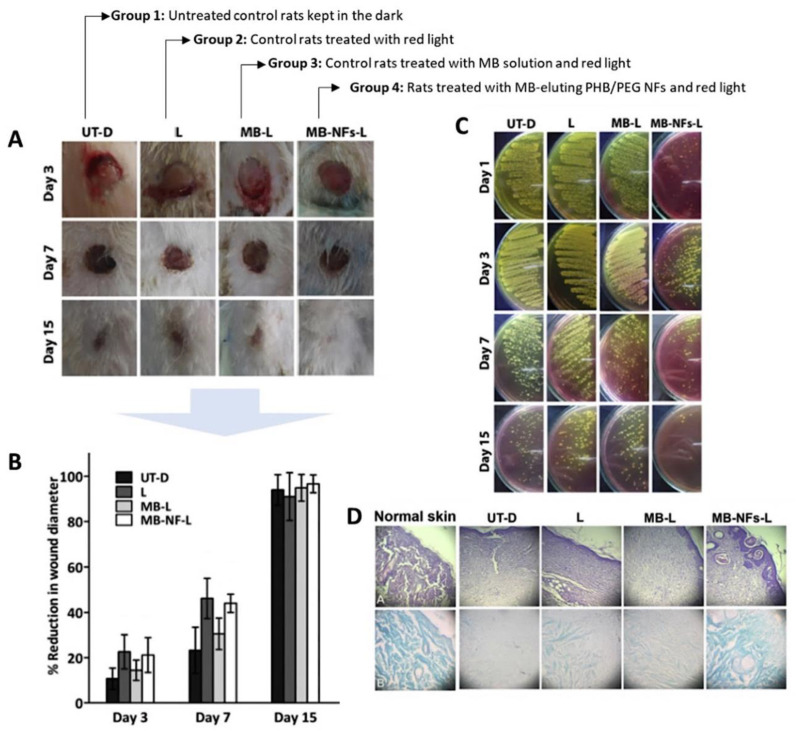
Healing evolution of different skin wound treatment groups. (**A**) Morphological characteristics of excision-infected wounds in different animal groups; (**B**) Time-course of percentage reduction in wound diameter; (**C**) Degree of wound bacterial contamination over 15 days; (**D**) Histopathological examination of H & E (top row) and Masson’s trichrome (bottom row) stained excision wounds after 15 days of wounding (figure adapted from [12] with permission of Elsevier).

**Figure 8:**
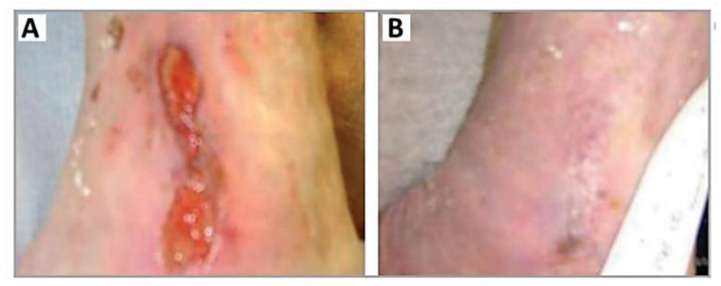
Evolution of a chronic leg ulcer (**A**) before and (**B**) 2 months after PDT treatment in the presence of the phenothiazinium-type photosensitizer PPA904 (figure adapted from [54] with permission of John Wiley and Sons).

In 2017, Khordagui and co-workers reported that MB incorporated in polymeric nanofibers (NFs) of polyhydroxybutyrate and polyethylene glycol (MB-NFs) [12] can improve the healing process for infected wounds induced in immunocompromised rats. In this promising study, the wounds inoculated with a *Staphylococcus aureus* strain (ATCC 6538P) were treated with MB-NFs and red light, and the results were compared with the ones obtained from adequate controls (Figure 7, Group 4 versus Group 1–3). The morphological, morphometric, microbiological and histological assays allowed to conclude that despite the progressive wound contraction in Groups 2–4 compared to control (Group 1), the wounds in Groups 2 and 3 remained heavily infected (Figure 7A and Figure 8C). These results indicate the inefficiency of the treatments mediated by light irradiation alone or by MB-aPDT to control the wound bacteria. Conversely, Group 4 (MB-NFs-aPDT) showed significant subsidence in wound infection up to Day 3 post-incision, and after 15 days, the wounds were completely healed, contrary to other groups (Figure 7B and Figure 8D). These results point out the importance of the interplay between the photosensitizer and the polymeric scaffold that aids the acceleration of the proliferative process, making this partnership important to consider for further developments related with the healing process for infected wounds not mediated by antibiotics. Nonetheless, the study lacks an adequate control group, using only the polymeric NF, to understand the isolated effect of the polymer in the wound.

#### 2.1.2. Toluidine O Blue Dye

Tolonium chloride, usually known as Toluidine O blue (TBO) (Figure 5), is another positively charged phenothiazine dye like MB. In 2009, Garcia et al. selected this PS to conduct a histological assessment of the PDT effect on repairing third-degree-burn wounds made on Wistar rats’ backs [53]. In the PDT assays, the activation of TBO (100 µg/mL applied 1 min before the light treatment) was conducted with red laser light at 685 nm, applied in continuous mode at nine points (power density of 0.5 W/cm^2^, with a total light dose of 40.5 J/cm^2^/point; full exposure time of 81 s). The results obtained from the aPDT group (Group 4) were compared with the ones obtained from burn lesions treated with laser red light alone (Group 3), burn lesions without any treatment (Group 2), and animals that were subjected to a cold punch and no treatment (Group 1). The wounds were monitored after 3 and 14 days. Only in Groups 3 and 4 was no chronic inflammation detected. Although the results seem to indicate that the difference between PDT (TBO plus irradiation at 685 nm) and low-level laser therapy (LLLT, 685 nm) in terms of the final achievements (e.g., re-epithelization scores, collagen fiber scores and hastening inflammation) is not significant, higher quality and better epithelium organization were observed in the PDT group. However, from the results obtained, it was not possible to conclude if the PS is advantageous for this treatment, when compared with the one using only low-level laser therapy.

#### 2.1.3. 3,7-Bis(*N*,*N*-Dibutylamino)phenothiazine-5-ium Bromide

In 2013, Morley et al. selected the MB analog 3,7-bis(*N*,*N*-dibutylamino)phenothiazine-5-ium bromide (Figure 5) to treat patients with leg ulcers and chronic diabetic foot ulcers (Figure 8) [54]. In this first controlled phase II study of antimicrobial PDT, the cationic dye, referred to by the authors as PPA904, was topically applied in the ulcers of 16 patients and activated with red light at 570–670 nm (50 J/cm^2^); the same number of patients that received only a placebo cream were used as the control group. Wound monitoring for 3 months revealed that there was better healing in areas that received the PS than those that only received the placebo cream (Figure 8). After 3 months, complete healing was observed in the leg ulcers of 50% (four of eight) of the patients compared with 12% (one of eight) of those receiving only the placebo treatment. The withdrawal of diabetic subjects with foot ulcers due to the administration of antibiotics did not allow the analysis of data. The authors concluded that besides the safety and simplicity of the treatment, no type of moderate or severe pain was detected. However, it was recognized that a higher number of patients was required to validate the approach, and further investment must be performed in the treatment protocol (the optimization of the drug formulation, light source and repeat treatments) to attain great bacterial cell kills and to assess wound healing.

#### 2.1.4. Benzophenothiazinium Dyes (EtNBS)

**Figure 9:**
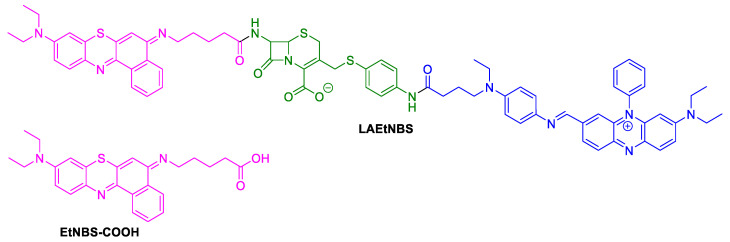
Structures of β-lactamase-activated EtNBS (LAEtNBS) and EtNBS-COOH.

In 2014, Fu evaluated the aPDT efficacy of the PS LAEtNBS (β-lactamase-activated EtNBS) (Figure 9) on wounds infected by MRSA or bioluminescent MRSA. This PS, constituted by a cephalosporin moiety coupled with a strong quenching molecule (BHQ-3) and with a benzophenothiazinium derivative EtNBS-COOH (Figure 9) on the terminal sites, was conceived to act after its activation by β-lactamase, specifically expressed by methicillin-resistant *S. aureus* (MRSA) [55].

**Figure 10:**
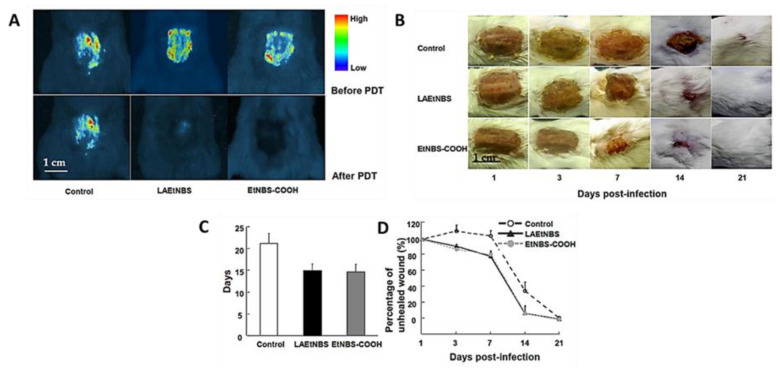
**(A**) Bioluminescent images taken 24 h after bioluminescent MRSA inoculation and 30 min after PDT with β-lactamase-activated benzophenothiazinium dyes (LAEtNBS), EtNBS-COOH and PBS (control); (**B**) Evolution of wounds in mice after treatment with PBS (control), LAEtNBS and EtNBS-COOH, respectively; (**C**) Mean time for complete wound healing in three groups; (**D**) Percentage of unhealed wound area in the three groups from Day 1 of the infection until wound healed (figure adapted from [55] with permission of Elsevier).

The results obtained after the PDT treatment (LED light at 90 J/cm^2^) of excisional wounds infected with the two MRSA strains performed in rat dorsa were compared with the ones obtained from controls: wounds treated with only EtNBS-COOH without light or only with PBS. Wound monitoring for 21 days allowed us to conclude that the animals subjected to PDT presented a gradually and satisfactory reduced wound area that was accompanied by the inactivation of bacteria (Figure 10). Statistical analysis indicated that PDT mediated by both LAEtNBS and EtNBS-COOH had a significant advantage for healing wounds infected by MRSA, but no significant difference was found between both PSs used, regarding the speed of wound healing. However, the authors believed that the modified PS based on β-lactamase targeting would be able to be applied in combination with standard antibiotic treatments to inactivate specific bacterial infections, particularly infections caused by multidrug-resistant bacteria. Nonetheless, the specificity of this PS was not explored since the authors did not assess the effectiveness of the treatment against different bacteria.

### 2.2. Naphthalimide Dye

Most of the studies related to the use of visible light combined with phthalimides dyes to catalyze the formation of bonds between tissues are related with meniscus restoration, articular cartilage and dura mater lesions, with an important contribution from Judy and co-workers’ studies [56,57,58,59].

**Figure 11:**
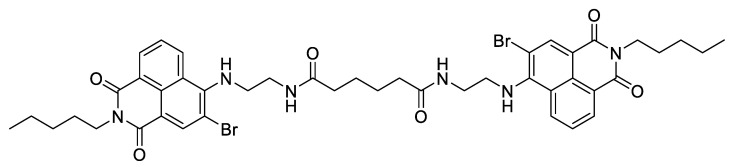
Structure of dibrominated 1,8-naphthalimide derivative dye.

In the context of skin lesions, the group reported, in 1998, an ex vivo study of photochemical bonding for wound closure, using the brominated dimeric l,8-naphthalimide dye (Figure 11) [60].

**Figure 12:**
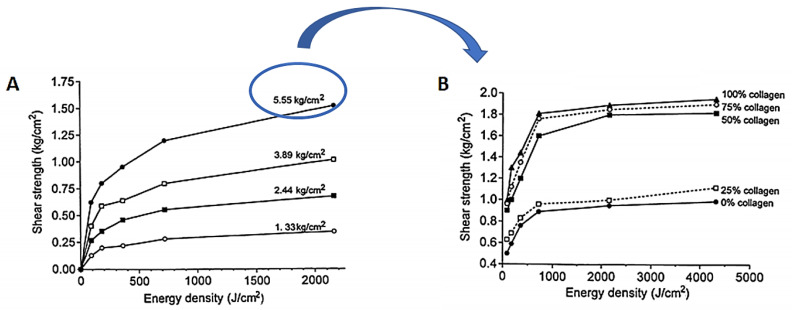
(**A**) Shear strength of rabbit dorsal skin exposed to different pressure values (1.33–5.55 kg/cm^2^) under blue light irradiation at 457.9 nm. (**B**) Effects of protein dosage on rabbit skin shear strength exposed to the selected pressure of 5.5 kg/cm^2^ and light exposure of 457.9 nm (figure adapted from [60] with permission of SPIE).

The study was performed using dorsal skin strips obtained from rabbits after removing the underlying hypodermis in order to leave a collagen-rich dermis. The results obtained after the irradiation of the PS applied on the bond surfaces, with an Argon laser light at 457.9 nm at an irradiance of 800 mW/cm^2^, showed an increase in the bond shear strength as a function of the externally applied pressure during the light exposure. The addition of suspensions of collagen–albumin (12 mg of total protein) to the PS also had a positive effect on the bond shear strength of the apposed and bonded strips (Figure 12). The bond strength without protein filler at 2 kJ/cm^2^ and 4 kg/cm^2^ external pressure was able to reach ca. 0.9 kg/cm^2^, while an increase in collagen from 25% to 100% attained a bond strength of 1.9 kg/cm^2^, under the same pressure and light exposure conditions.

The histological examinations of the bonded specimens were, in general, like the normal dermis, and therefore, it was concluded that these promising results with 1,8-naphthalimide dyes and collagenous fillers must be extended to in vivo studies.

### 2.3. Indocyanine Green

**Figure 13:**
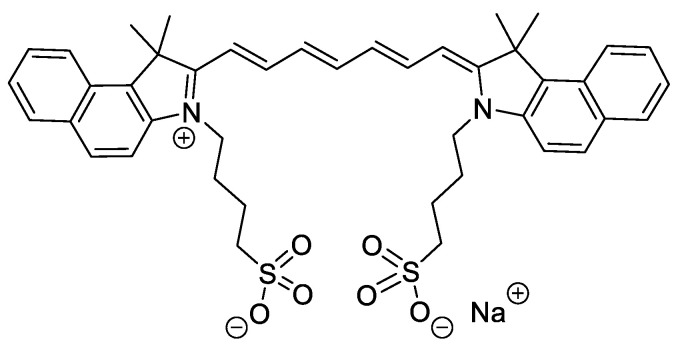
Chemical structure of indocyanine green.

Indocyanine green (ICG) (Figure 13) is a dye approved by the Food and Drug Administration (FDA) and is being considered particularly attractive for different medical purposes (e.g., medical imaging, acne vulgaris and ophthalmological treatments) due to its low toxicity [61,62,63,64,65]. Its anionic nature limits its use as an antibacterial photosensitizing agent. However, this drawback is, in a certain way, compensated by its highly intense absorption band around 800 nm, allowing its use to treat deep infections since the light penetration at 800 nm in tissues is almost twice that with visible light [66].

**Figure 14:**
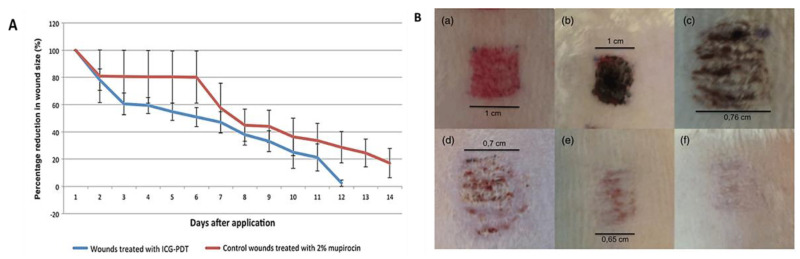
(**A**) Percentage of the decrease in wound size with (blue) and without (red) PDT treatment, monitored for 14 days; (**B**) Physical appearance of the wound without (a) and with PDT-treatment at Day 0 (b), Day 1 (c), Day 4 (d), Day 7 (e) and Day 11 (f) (figure adapted from [67] under Creative Commons (CC BY 4.0)—Gold Open Access).

In 2015, Topaloglu et al., based on their in vitro achievements in the lethal photosensitization of strains of *S. aureus* and *Pseudomonas aeruginosa* after the activation of ICG at 809 nm, reported the first in vivo study using this PS to treat abrasion wounds infected with *S. aureus* in rats [67]. This low-bleeding lesion was selected in order to minimize the interaction of ICG with plasma proteins and, consequently, ensure its ability to absorb enough light to produce the required ROS to photoinactivate bacterial cells. A decrease of around 90% in bacterial burden was observed in wounds subjected to PDT treatment (ICG, λ_exc_ = 808 nm; total light dose of 450 J/cm^2^; 15 min of irradiation), while no significant diminishing was observed in the positive controls (ICG and laser groups). The study also showed that the healing process in PDT-treated wounds was much faster than that in the 2% mupirocin-treated wounds; at Day 11, the sizes of the PDT wounds approached zero, while the 2% mupirocin-treated wounds were still visible after 14 days (Figure 14). According to the authors, the histological analysis was in accordance with these findings, since, in PDT-treated wounds, the epithelial lining reached its normal thickness, the epidermis integrity was uniform, and fewer fibroblasts were observed.

The effect of PDT on cystic fibrosis transmembrane conductance regulator (CFTR), an ion channel expressed in multiple layers of keratinocytes that helps to induce wound healing, was evaluated, in 2019, by Chiu and coworkers, also using ICG as a PS [68]. In this study, the PS was applied topically in mouse wounds and then irradiated with infrared light at 780 nm with an irradiance of 65.5 mW/cm^2^ and exposed to a total light dose of 5 or 15 J/cm^2^. The wounds were monitored for 15 days, and it was observed that wounds exposed to a total light dose of 5 J/cm^2^ healed faster than those exposed to a total light dose of 15 J/cm^2^. Additionally, in vitro, it was observed that 5 J/cm^2^ ICG-PDT improved cell migration, whereas 15 J/cm^2^ ICG-PDT inhibited cell migration. Similar effects were observed in mouse skin wound healing, leading the authors to conclude that values below 15 J/cm^2^ must be used to achieve the best healing results with this PS. A possible justification for this enhancement may be related to the increase in the CFTR activation, responsible for the recruitment of other molecules, such as focal adhesion kinase (FAK) and paxillin, that are important for promoting cell proliferation.

### 2.4. Rose Bengal Dye and the Concept of Photobonding

**Figure 15:**
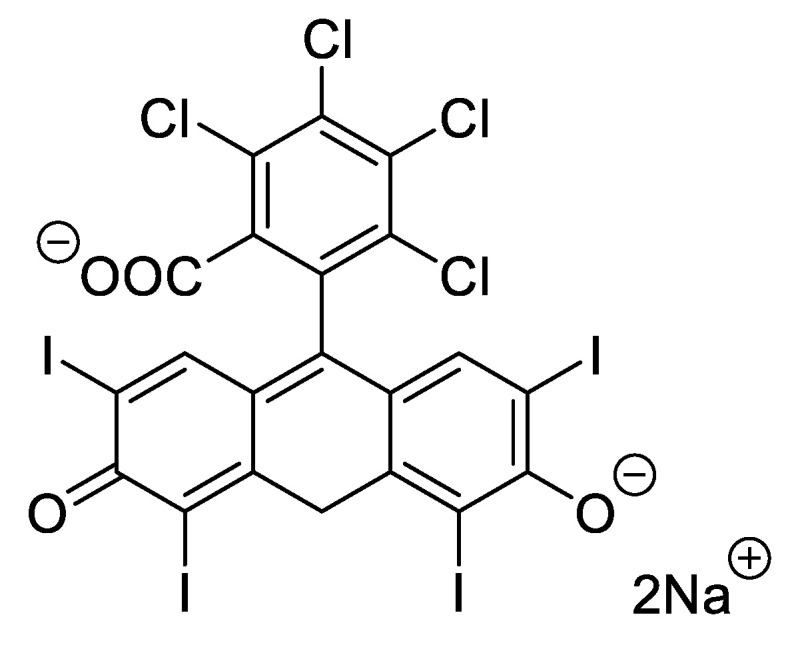
Structure of Rose Bengal (RB) dye.

4,5,6,7-tetrachloro-2′,4′,5′,7′-tetraiodofluorescein, usually known as Rose Bengal (RB) (Figure 15), is also earning some attention from researchers due to its safety (e.g., it is used as a systemic hepatic and topical ophthalmic diagnostic agent) and lack of cutaneous toxicity and phototoxicity at low irradiances [41].

**Figure 16:**
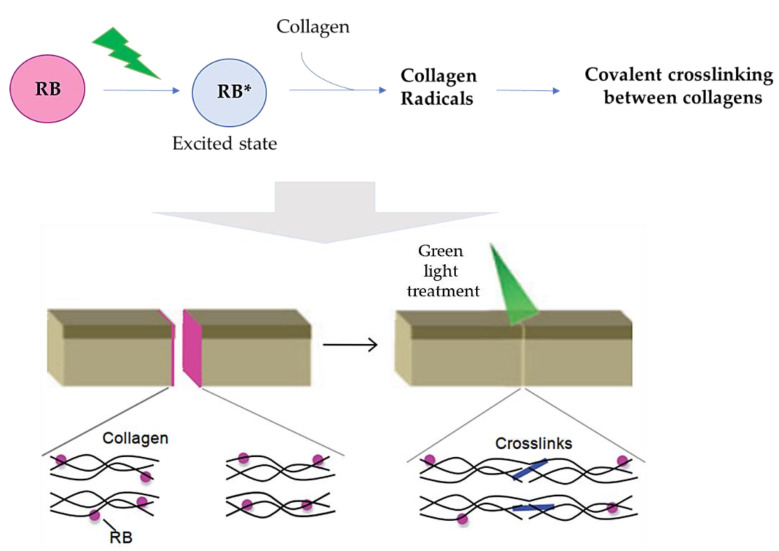
Schematic representation of the proposed mechanism for wound healing promoted by RB (figure adapted from [42] with permission of John Wiley and Sons).

According to some authors, RB, when applied onto wound surfaces, is able to be associated with collagen and to initiate, after its light activation, a series of electron transfer reactions involving excited RB and some amino acids present in proteins. When this phenomenon occurs on the apposed tissue of a wound, the coupling of ended protein radicals is responsible for covalently crosslinking collagen polymeric chains, which consequently allows tissue adherence (Figure 16) [42,69,70,71]. This process is often referred to as photobonding (PB).

**Figure 17:**
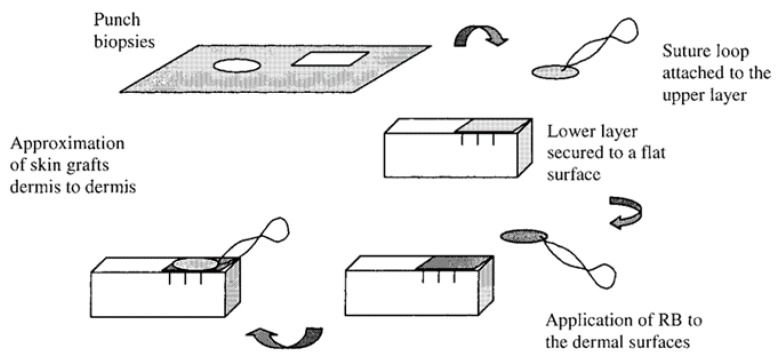
Preparation of skin samples. Two pig skin samples with the photosensitizer in the middle were put in contact, and then, the photobonding was promoted (figure adapted from [71] with permission of Elsevier).

In 2002, the research group of Kochevar studied the potential of the RB dye to improve porcine skin graft adherence after irradiation with green light at 514 nm [71]. In this ex vivo study, the RB in PBS was applied in two skin grafts for 2 min before the irradiation protocol (514 nm at an irradiance of 0.56 W/cm^2^ for 15 min and a total light dose of 504 J/cm^2^). The results showed that adherence between the grafts was dependent on the RB concentration, with a plateau at 0.1% (*w*/*v*). This dose dependence is consistent with a photochemical mechanism involving the reactive species and the crosslinkable targets in the nearby tissues. It was suggested that the effect of higher RB concentrations can promote the formation of inactive aggregates and the self-quenching of reactive species by the dye. The saturation of crosslinkable sites at the dermis surface can also decrease the bonding efficiency. In the presence of 0.1% RB, it was also observed that the graft adhesion increased with the total light dose up to 504 J/cm^2^. The results obtained indicated that the photobonding approach has a great potential to mediate the linkage between skin grafts since no significant thermal effects were detected and the skin viability was not compromised. However, it was recognized that further in vivo studies are necessary to ensure the effectiveness of this approach as an alternative to sutures and also to determine the extent of scar tissue formation as a function of post-grafting time (Figure 17).

**Figure 18:**
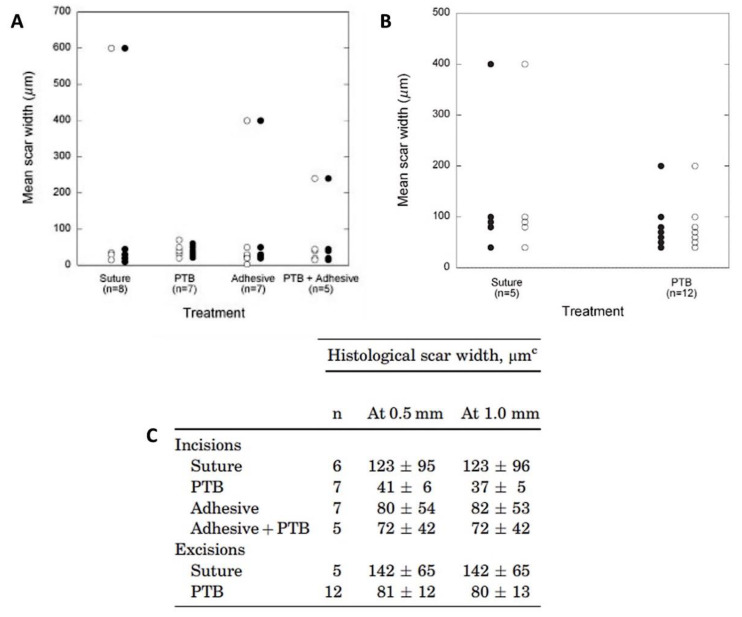
Results for scar widths of wounds treated with the different procedures. (**A**) Comparison of histological widths of scars from excisions closed with sutures or photoactivated tissue bonding (PTB), and (**B**) comparison of histological widths of scars from the different incisions. Open circles denote measurements made at 0.5 mm from the skin surface, and closed circles denote measurements made at 1.0 mm; (**C**) Histological scar widths 6 weeks after the closure of incisions and excisions (figure adapted from [70] with permission of John Wiley and Sons).

Considering the promising results obtained in the previous ex vivo studies, the same research group evaluated, in vivo, the efficacy of RB in repairing incision and excision wounds [70]. The wounds were performed in a pig model, and the photobonding results were compared with conventional suture and tissue adhesive treatments. The irradiations were performed at 532 nm at an irradiance of 0.56 W/cm^2^ using a Nd:YAG laser. After 6 weeks, both the “mean scar width” and “histological scar width” measured for the incisions showed no significant differences between the group that received the photoactivated tissue bonding (PTB) (laser + RB; total light dose of 100 J/cm^2^) and the controls (only sutures; adhesive (Dermabond^TM^; laser + RB + adhesive with total light dose of 100 J/cm^2^) (Figure 18). A similar conclusion was found for the excision wounds (irradiated and receiving different light doses of 75, 100 or 150 J/cm^2^). However, the scar widths were slightly smaller in the wounds treated with PDT. Thus, the authors considered that the potential of photobonding to close incisions and excisions with minimal scar development must be further explored in human skin.

**Figure 19:**
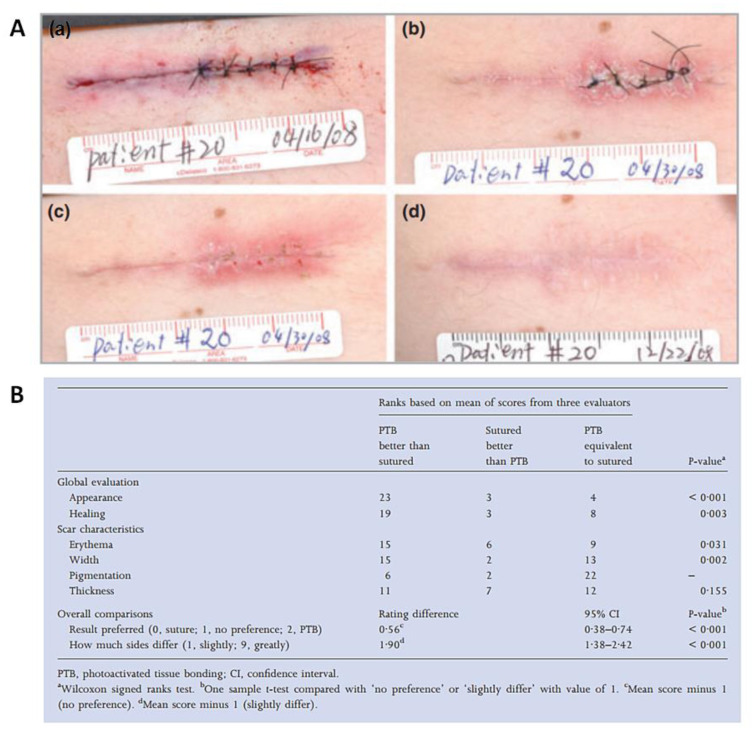
(**A**) Epidermal closure of a skin excisional wound half treated with suture and half with PB: immediately after superficial closure (a), at Day 0 (b), at 2 weeks post-surgery (c) and at 6 months post-surgery (d); (**B**) Classification of the excision sites after 6 months, resulting from the evaluation of three dermatologists, using the POSAS (Patient and Observer Scar Assessment Scale) (figure adapted from [42] with permission of John Wiley and Sons).

In this context, in 2012, the group reported promising outcomes from the first human study where PB also mediated by RB was used in wound closure [42]. The study involved twenty-seven patients with thirty-one skin lesions in different parts of the body (e.g., the chest, abdomen or arms), and the results from the photoactivated tissue bonding treatment were compared with the ones using standard epidermal suturing. In the photobonding approach, the RB solution was applied topically in the wound and then irradiated with a green light at 532 nm, with a total light dose of 100 J/cm^2^ (3 min of irradiation). The wounds were monitored for 6 months, and it was observed that the wounds were successfully closed, with less fibrosis and with superior scar appearance than with closure with conventional interrupted sutures (Figure 19A). Three evaluators scored the images of the excision sites treated with photobonding, compared with suture, and the results were also in favor of the PS treatment (Figure 19B).

**Figure 20:**
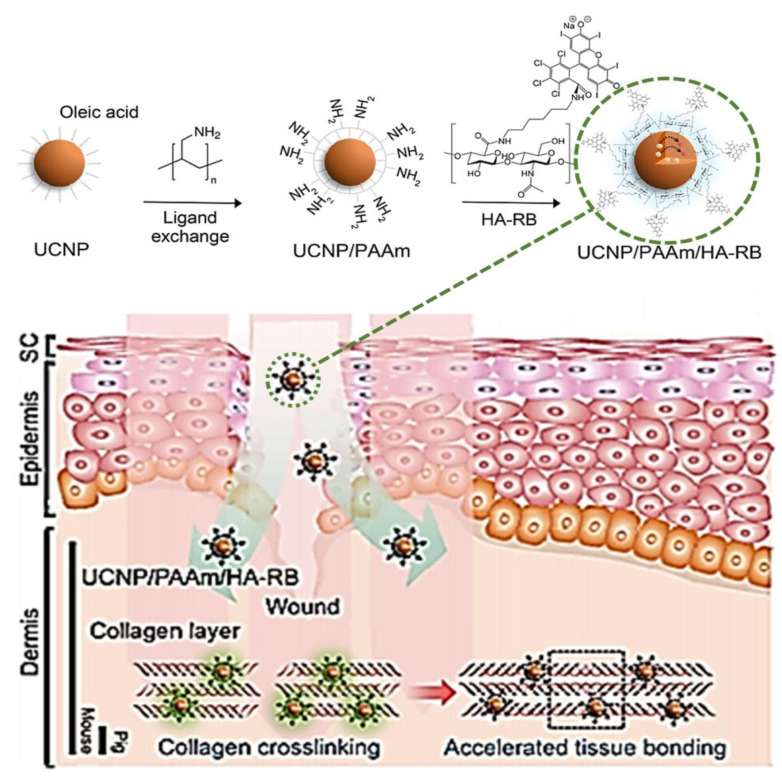
Synthesis of upconversion nanoparticles (UCNPs)/poly(allylamine) (PAAm)/hyaluronate (HA)-RB conjugate, and schematic illustration of their application in transdermal delivery (figure adapted with permission from [72]. Copyright (2020) American Chemical Society).

Han et al. reported the development of the RB conjugate UCNP/PAAm/HA-RB (Figure 20) and evaluated its efficacy in accelerating sutureless tissue bonding [72]. The access to the conjugate required the synthesis of upconversion nanoparticles (UCNPs) modified with poly(allylamine) (PAAm), UCNP/PAAm, and of the hyaluronate (HA) adduct of RB, HA-RB (Figure 20). The electrostatic interaction between UCNP/PAAm and HA-RB conjugate gave rise to the desired conjugate (Figure 20) that was applied in mouse dorsal skin incisions. The irradiations were performed for 20 min using an infrared laser at 980 nm at an irradiance of 500 mW/cm^2^ or green light at 540 nm with an irradiance of 7.5 mW/cm^2^, and the wound evolution (monitored for 3 days) was also compared with the evolution of incisions not subjected to any treatment. The results obtained showed that the incisions treated with the conjugate UCNP/PAAm/HA-RB and infrared laser had the fastest tissue bonding, followed by the group where the conjugate was irradiated with green light and then by those only irradiated with infrared light. The authors believe that the success of the conjugate in mediating this noninvasive photo tissue bonding is related to a better penetration of RB in the incision that may be facilitated by HA and, concomitantly, by the ability of the nanoparticles to guide light into the deep tissue (Figure 20).

**Figure 21:**
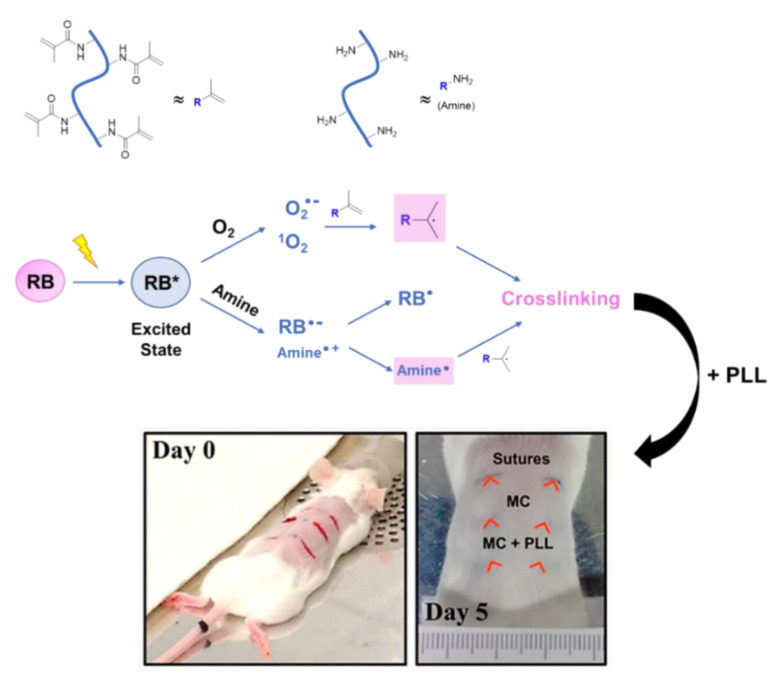
Schematic representation of reactive intermediates formed upon RB irradiation, leading to polymeric matrix crosslinking responsible for the ultimate tissue photobonding. Picture: Left—Representative image of the surgical incisions made on the mouse’s back skin before treatment. Right–Representative image after 5 days of treatment (figure adapted with permission from [73]. Copyright (2020) American Chemical Society).

In 2017, Pupkaite et al., in the same context of developing materials based on RB for sutureless wound closure, reported the incorporation of this PS in hydrogels of methacrylate-modified type I collagen (MC), followed by its embedment in poly-*L*-lysine (PLL) (Figure 21) [73]. With this formulation, it was envisioned to combine the collagen hydrogel formation ability with the potential of RB for crosslinking it (Figure 21). With the presence of PLL, the authors tried to decrease the formulation’s degradation rate and to improve its mechanical properties (Young’s modulus and breaking strain analysis). The biological results showed that mouse incisions treated with this new formulation and then irradiated with a green LED light at 523 nm with an irradiance of 220 mW/cm^2^ for 10 min (total light dose of 132 J/cm^2^) had a better cosmetic appearance than the ones subjected to traditional sutures or treated only with the methacrylate-modified type I collagen used as control (Figure 21). In fact, after 5 days, the wounds receiving hydrogels closely resembled non-wounded skin, whereas the sutured group showed signs of erythema, suggesting inflammation. From the histological evaluation, the authors concluded that the control group (only treated with methacrylate-modified type I collagen) appeared to have the underlying dermis invaded by erythrocytes/monocytes, with a more disorganized, fibrous look. These results clearly show that this approach may be a viable alternative to surgical stitching. Regarding the mechanical properties, the authors reported that the Young’s modulus of tissues bonded with the MC-RB formulation was significantly lower than that obtained with sutures, whereas the breaking strain of photobonded tissue was similar to the sutured control.

**Figure 22:**
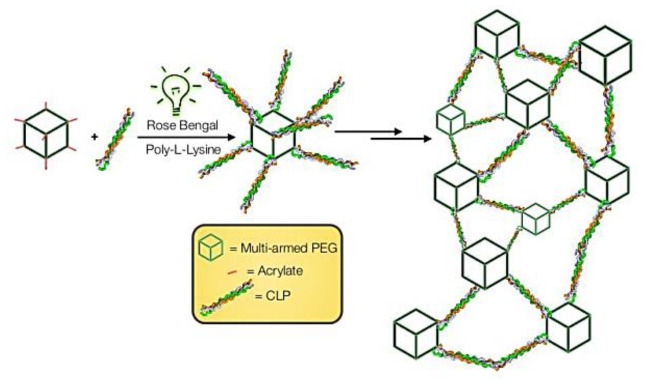
Schematic illustration for light-triggered assembling of a photocrosslinkable adhesive hydrogel material (figure adapted with permission from [69]. Copyright (2020) American Chemical Society).

Two years later, the same group contributed with another interesting study concerning the potentiality of RB in PB [69]. In this study, the authors developed an adhesive hydrogel using, as monomers, collagen-like peptides (CLP) conjugated to a single-arm polyethylene glycol-acrylate (PEG-Ac) and a multi-arm PEG-Ac. The copolymerization was performed in the presence of RB and PLL, which acted as both an electron donor and an active participant in the polymerization process for the hydrogel (Figure 22). The performance of these materials in wounds was evaluated after their irradiation with a green LED light (at 532 nm at an irradiance of 3.3 mW/cm^2^ for 15 min with a total light dose of 2.97 J/cm^2^), and the results obtained were compared with the ones from the classical suture used as a control. All wounds were closed after 7 days, however, the PS-mediated wound closure promoted a faster epithelial regeneration and diminished scar tissue formation compared to the control wound.

### 2.5. Other Photoactive Platforms

The diversity of systems with photocatalytic features is responsible for other contributions in the context of combining wound healing with PDT protocols. In this section, some of those promising achievements comprising not only organic dyes but also platforms containing phosphorous nanosheets and photoactive hydrogels doped with inorganic nanoparticles are discussed.

**Figure 23:**
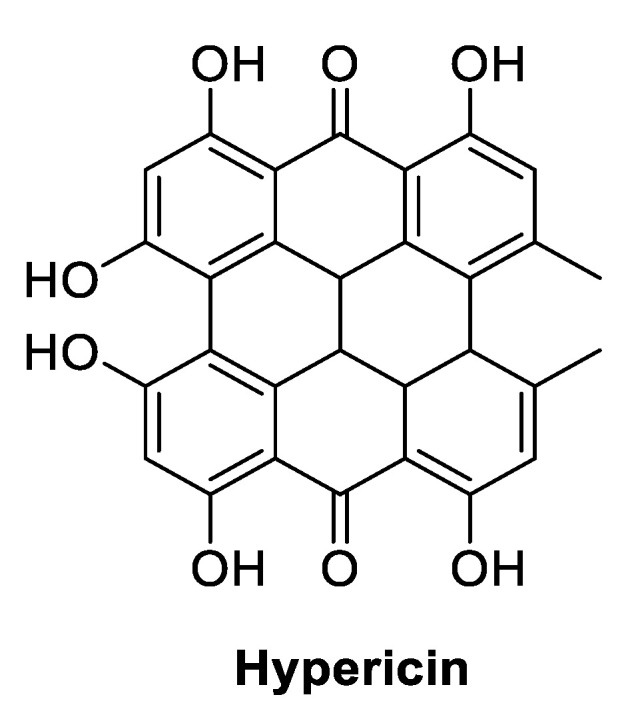
Chemical structure of hypericin (HY) used as PS in wound healing PDT-assisted studies.

Among the organic dyes is hypericin (HY) (Figure 23), a polycyclic-based anthraquinone metabolite extracted from the St. John’s Wort (*Hypericum perforatum*) plant, used as a wound healer in antique times and with recognized antimicrobial activity towards several bacterial and fungal strains [74]. The antimicrobial activity of HY is associated with the presence of light, mainly in organic solvents [75,76].

**Figure 24:**
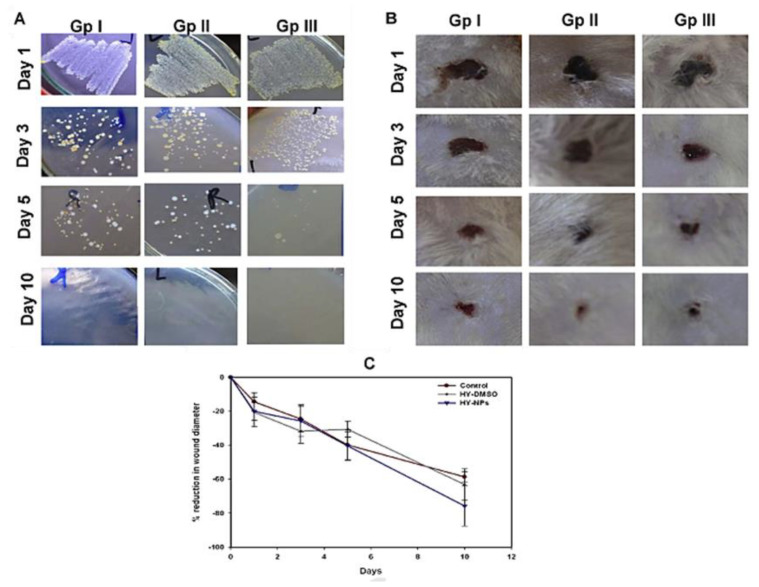
Influence of HY-NPs in the healing of rat infected wounds. (**A**) Antibacterial effect of HY-NPs against an MRSA strain; (**B**) evolution of infected wound thickness (GP I—untreated control; GP II—free HY in DMSO; GP III—HY-NPs); (**C**) reduction of wound diameter. The wounds were monitored for 10 days (figure adapted from [77] with permission of Elsevier).

Aiming to overcome the lipophilicity limitation of HY for therapeutic applications, Nafee and coworkers reported, in 2013, the development of a biodegradable nanocarrier, where the antibacterial photodynamic action of HY was preserved and, concomitantly, promoted a positive effect in wound healing [77]. The authors selected the amphiphilic di-block copolymer polycaprolactone-poly(ethylene glycol) (PCL-PEG) for nanoparticle preparation, due to the slow degradation of PCL via enzymatic degradation or the hydrolysis of ester bonds. The copolymer was synthesized by the ring-opening polymerization of caprolactone monomer in the presence of poly(ethylene glycol)monomethyl ether (m-PEG) and using tin(II) 2-ethylhexanoate as a catalyst. The ability of HY nanoparticles (HY-NPs) with a diameter of ca. 45 nm (obtained by a coprecipitation approach using a PCL-PEG copolymer in the presence of HY) to generate reactive oxygen species upon irradiation was confirmed. Additionally, the in vitro studies, performed against biofilm formation and planktonic cells of MRSA clinical isolates, demonstrated a superior inhibition of biofilm formation over planktonic cell growth. The healing potential of HY-NPs after irradiation with white light and a total light dose of 23.5 J/cm^2^ (Group III) was evaluated in rat wounds infected with MRSA, and the results were compared with the ones obtained from non-treated wounds (Group I) or wounds treated only with free hypericin and the light irradiation protocol (HY-DMSO) (Group II). The treatments were carried out for five consecutive days, and the wounds were monitored for 10 days. The results reflected a gradual healing improvement in all the groups. However, there was an obvious improvement in the healing rate for the groups under PDT treatment (Figure 24B,C). In vitro antibacterial assays against MRSA also showed that PDT treatment was effective in eliminating almost all bacteria after 5 days, contrarily to what happened in the control groups (Figure 24A). Interestingly, the survival rates of the rats were 50%, 75% and 100% for Groups I, II and III, respectively, throughout the whole study period. The study also showed a better epithelialization and keratinization of skin layers for wounds treated with HY-NPs.

**Figure 25:**
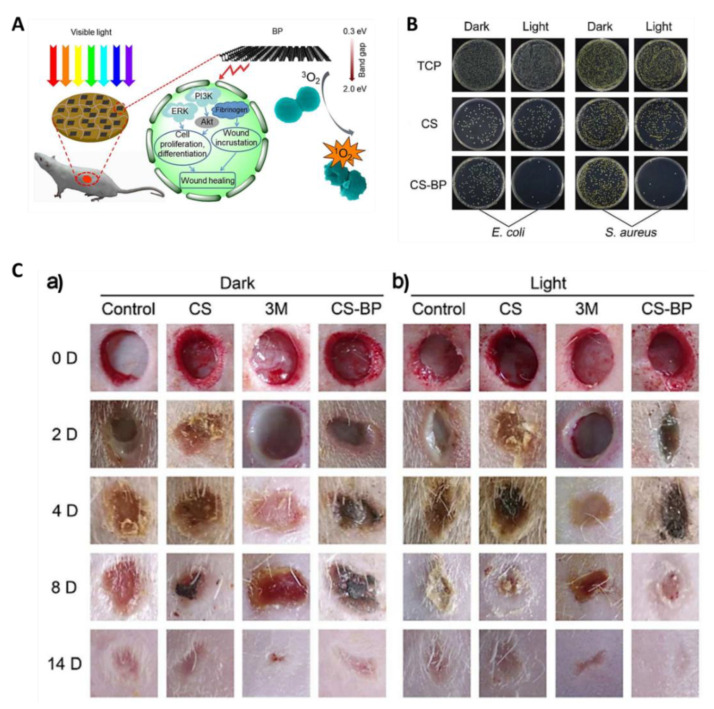
Influence of chitosan (CS)–black phosphorus (BP)-based hydrogels in rat infected wound healing. (**A**) Schematic illustration of visible light sterilization that stimulates skin regeneration in the presence of CS/BP nanosheet-based hydrogel; (**B**) *E. coli* (left) and *S. aureus* (right) colony units in dark conditions and after PDT for 10 min under simulated sunlight. The control tissue culture plate (TCP) was compared with CS- and CS-BP-based hydrogels; (**C**) Healing effect in *S. aureus*-infected wounds after in vivo treatment under dark conditions (a) and simulated sunlight for 10 min at Days 0, 2, 4, 8 and 14 (b) (figure adapted with permission from [80]. Copyright 2020 American Chemical Society).

The ability of ultrathin 2D nanosheets obtained from the exfoliation of black phosphorus (BP) to produce ROS [78,79] prompted the development of a hydrogel based on the electrostatic interaction between chitosan (CS) and BP nanosheets (CS-BP) (Figure 25A) [80]. The synthesis of this hydrogel CS-BP required only the embedment of previously prepared CS hydrogel wafers into a BP aqueous solution for 10 min. Figure 25B shows that, in the presence of a CS-BP-based hydrogel, an almost-full photoinactivation for *S. aureus* and *Escherichia coli* bacteria was observed, while CS induced lower antibacterial reduction ratios (70% for *E. coli* and 60% for *S. aureus*). In turn, in vivo studies showed that the infected wounds in rat skin subjected to irradiation with white light (a 200 W xenon lamp) for 10 min in the presence of CS-BP were significantly healed after 14 days, when compared with the controls “3M” (commercial wound dressing) and CS, indicating that the rapid sterilization mediated by ^1^O_2_ played a crucial role in wound healing (Figure 25C). These results are in agreement with the improvement also observed in the formation of fibrinogen at the early stages of the tissue repair process and in the activation of ERK1/2, PI3K and Akt kinases that are responsible for cellular proliferation and signaling pathway differentiation during tissue reconstruction.

**Figure 26:**
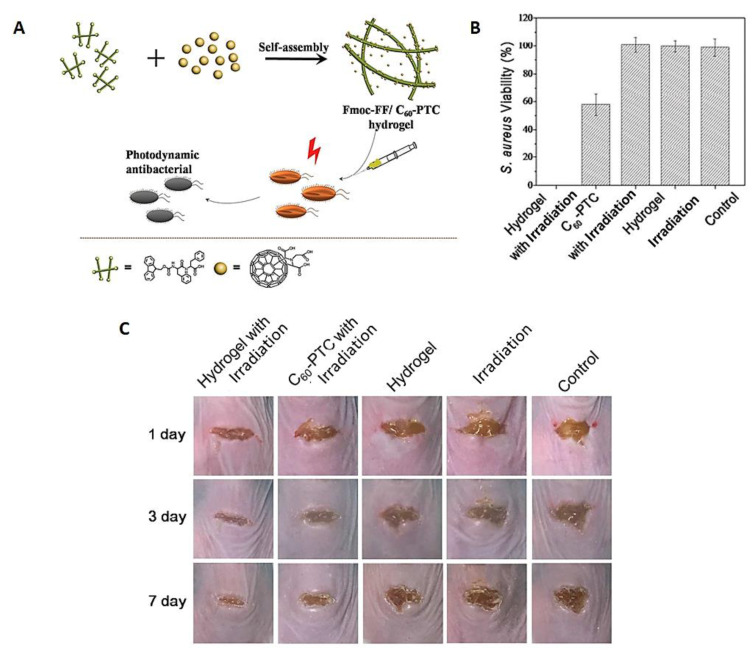
Influence of Fmoc-FF/C60-PTC-based formulation on *S. aureus* growth and mouse infected wound healing. (**A**) Self-assembling process to prepare Fmoc-FF/C60-PTC-based formulation; (**B**) *S. aureus* colony viability; (**C**) Photographs of mouse wound after 1, 3 and 7 days of antimicrobial PDT (aPDT) treatment (figure adapted from [81] with permission of Royal Society of Chemistry).

Knowing that the biomedical applications of fullerenes are limited by their low aqueous solubility and an apparent tendency to undergo aggregation, in 2018, Zhang et al. reported the development of an injectable hydrogel formulation obtained by self-assembling the small peptide *N*‑fluorenylmethoxycarbonyl diphenylalanine (Fmoc-FF) with C_60_ pyrrolidine tris-acid derivative (C_60_-PTC) and evaluated its efficacy in multidrug-resistant *S. aureus* inhibition and mouse infected wound healing (Figure 26A) [81]. The formulation was obtained by pouring water into a mixture containing both components, the C_60_-PTC dissolved in *N*,*N*-dimethylformamide (DMF) and the Fmoc-FF in DMSO under sonication. After aging and dialysis to remove the organic solvents, the characterization of the obtained peptide nanofibers confirmed the presence of small nanoparticles of fullerenes and an improvement in ^1^O_2_ generation activity, when compared with C_60_-PTC. In vitro studies using this formulation showed a better performance of the hydrogels under irradiation in the elimination of *S. aureus*, when compared with the non-formulated C_60_-PTC control (Figure 26B).

Additionally, the better efficacy of the hydrogel formulation was also patent when it was assessed in infected wounds created in mice and irradiated under white light at an irradiance of 100 mW/cm^2^ for 10 min with a total light dose of 60 J/cm^2^ (Figure 26C). The outcome was compared with the results obtained from the control groups [(non-formulated C_60_-PTC precursor subjected to the same light protocol (C_60_-PTC with irradiation), wounds treated with the hydrogel formulation but in the absence of light (hydrogel), wounds treated only with white light (irradiation), or no treatment (control)]. The results revealed that at the third observation day, the mouse wounds treated by PDT were partly healed, while the wounds of the mice control groups showed low or no improvement. One week later, the wounds treated by PDT were almost healed, whereas the other mouse groups were still in an infected state. The results seem to indicate that the combination of the hydrogel formulation and the PS features provided the wounds with physical conditions for healing more properly than the control groups. The effectiveness of the Fmoc-FF C60-PTC formulation in combating the multiresistant *S. aureus* and promoting wound healing offers a promising paradigm for adapting the self-assembling small peptides with the integration of multiple functions for biomedical applications.

**Figure 27:**
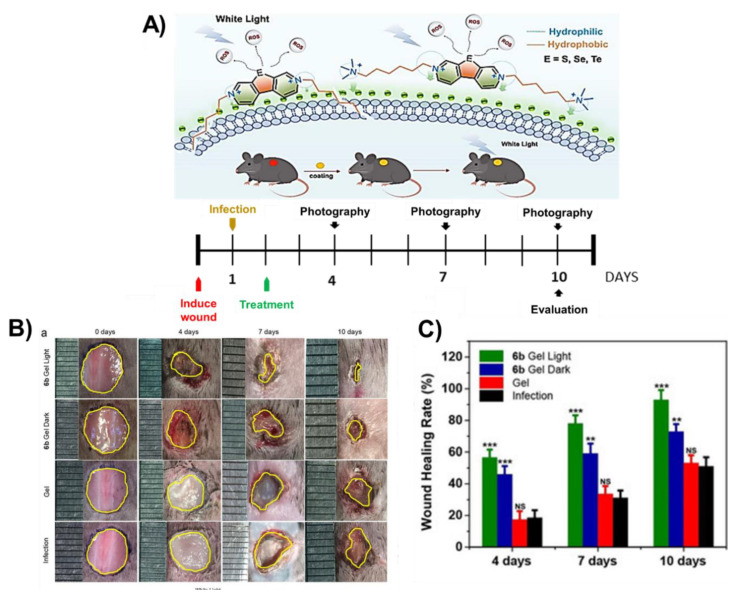
(**A**) Schematic mode of action of the photosensitizer in wound healing; (**B**) photographs of wound regions from the different treatment groups, at the timepoints indicated; (**C**) wound healing rates for the different studied groups (figure adapted from [82] with permission of Royal Society of Chemistry).

In 2019, Zhou et al. developed a series of cationic chalcogenoviologen derivatives (Figure 27) with different heteroatoms atoms (S, Se and Te), bearing hydrophilic or hydrophobic lateral chains, and found that the best PS acting against *S. aureus* and *E. coli* growth was a selenium derivative with a hydrophobic C_6_ chain [82]. This fact prompted the authors to evaluate the efficacy of this selenium derivative after being loaded in a hyaluronic acid hydrogel patch (6b Gel) to treat mouse infected wounds (Figure 27) [82]. After irradiation with white light, the wounds treated with the “6b Gel” showed an area significantly smaller (related with a faster healing rate) than the non-treated wounds, or the ones only treated with hyaluronic acid gel or with the “6b Gel” but without light irradiation (Figure 27A–C). The authors also had the chance to monitor the wound neovascularization and observed that the PDT-treated wounds produced far more microvessels, indicating that an effective bacterial suppression was crucial for the re-establishment of a new blood supply. All these results allowed the conclusion that the PS antibacterial properties played an important role in the skin regeneration process, and the PS success was associated with (1) the extended visible light absorption due to the presence of chalcogen atoms and the consequent improvement in ROS generation, and (2) the cationic backbones shortening the interaction distance with bacteria, leading to an efficient ROS delivery and bacteria death.

Silver is also appearing as an important partner in the development of some platforms, with a combined action of antimicrobial activity/wound healing due to localized surface plasmon resonance and its well-known intrinsic antimicrobial activity. Besides, titanium dioxide (TiO_2_) is also being considered an inexpensive and attractive antimicrobial derivative due to its photocatalytic nature and chemical stability and it is generally recognized as safe substance (GRAS). Thus, promising hydrogels were developed considering the combination of TiO_2_ with silver nanoparticles. Briefly, after Ag/TiO_2_ irradiation with visible light, a charge transfer to Ag nanoparticles suppresses the electron–hole recombination, and reactive oxygen species can be formed by transfer to molecular oxygen or by water’s loss of a proton to form hydroxyl radicals. Using this elegant platform design, the photocatalytic activity of TiO_2_ was extended to the visible region, while the toxicity of silver nanoparticles was reduced due to hydrogel entrapment [83].

**Figure 28:**
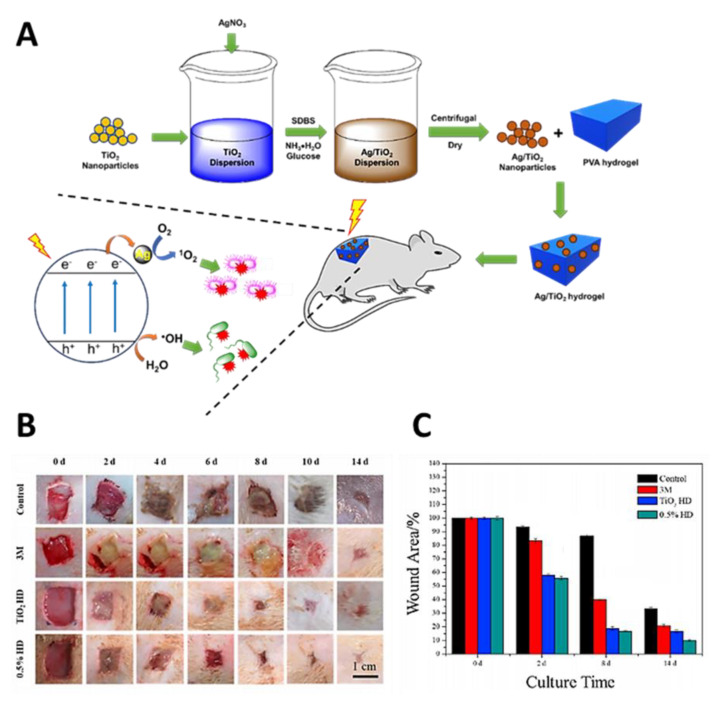
Influence of Ag/TiO_2_ nanoparticle hydrogel on mouse infected wound healing. (**A**) Ag/TiO_2_ nanoparticle hydrogel preparation and ROS production under red light irradiation at 660 nm; (**B**) photographs of wound regions of control, 3 M, TiO_2_ HD and 0.5% HD groups under light irradiation at 660 nm; (**C**) Percentage of wound area over 14 days of treatment) (figure adapted from [83] with permission of Elsevier).

Hydrogels based on poly(vinyl alcohol) (PVA) containing Ag-doped TiO_2_ nanoparticles were prepared using a simple two-step process (Figure 28A) [83]. The procedure involved the addition of the desired amount of AgNO_3_ to TiO_2_ nanoparticles dispersed in deionized water with sodium dodecylbenzenesulfonate (SDBS), followed by the chemical reduction of Ag^+^ in the presence of glucose. The obtained Ag-doped TiO_2_ nanoparticles with different contents of silver (0.2, 0.5, 1, 2 and 5%) were then dispersed in PVA using repeated freeze–thaw cycles (five times). The study showed that the hybrid with 0.5% Ag (0.5% hydrogel), besides its excellent antibacterial activity (under red light irradiation at 600 nm; 0.6 W/cm^2^ for 5 min) against both *E. coli* and *S. aureus* caused by ROS release, also possessed excellent biocompatibility. In addition, from the in vivo assays, it was found that these Ag-doped TiO_2_ nanoparticle hydrogels accelerated the healing in *S. aureus*-infected wounds, when compared with a traditional 3M dressing (Figure 28B,C), with no appreciable toxicity to organs.

Other hydrogels embedded with Ag/Ag@AgCl/ZnO nanostructures were also reported as effective composites for mitigating bacterial infections and accelerating wound healing after irradiation with visible light [84]. In this approach, the authors envisaged that the appropriate amount of Ag could improve the low antibacterial activity of ZnO (associated with inefficient photoenergy conversion due to low charge separation and the rapid recombination of the charge carrier), due to its role in enhancing the formation of ROS mediated by visible light irradiation. On the other hand, it was expected that the Zn(II) ions released from ZnO could promote the production of fibroblasts, which are particularly important during skin regeneration due to their differentiation into myofibroblasts in the dermis and subcutaneous tissues surrounding the wound. The authors selected a natural and toxicologically innocuous polysaccharide, carboxymethyl cellulose (CMC), to prepare the composites using a UV-light chemical reduction process to assemble the Ag/Ag@AgCl nanostructures, followed by the incorporation of ZnO nanostructures by NaOH precipitation. After confirming, in vitro, the antibacterial efficiency of the hydrogel composites (obtained with different initial concentrations of AgNO_3_—0.75, 1.25 and 2.5 mM) upon visible light exposure for 20 min against both *E. coli* and *S. aureus* (in vitro inactivation of ca. 95.95% for *E. coli* and 98.49% for *S. aureus*), their therapeutic efficacy in animal models with infected wound healing was evaluated and compared with adequate controls (pure CMC hydrogel, Ag/Ag@AgCl, and pure ZnO). The results showed that all the hydrogel composites with silver showed a smaller trauma size than the control group after 8 days of treatment, especially with Ag/Ag@AgCl/ZnO. The best antibacterial effect was also observed with the hydrogel composite Ag/Ag@AgCl/ZnO, where only a few viable bacterial colony units were formed. After 14 days of treatment, the wounds treated with both Ag/Ag@AgCl/ZnO and pure ZnO showed significant healing when compared with the control and Ag/Ag@AgCl groups. The synergistic antibacterial effect and accelerated wound healing were associated with the immune function stimulation to produce white blood cells and neutrophils (2–4 times more than the control) caused by Ag^+^ and Zn^2+^ release [84].

**Figure 29:**
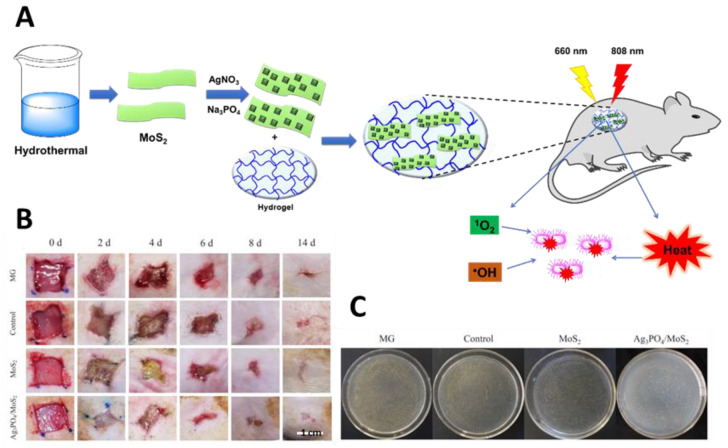
Influence of Ag_3_PO_4_/MoS_2_ hydrogel composite in the healing of rat wounds infected with *S. aureus*; (**A**) Schematic representation of Ag_3_PO_4_/MoS_2_ hydrogel preparation and synergic actuation of PDT and photothermal therapy (PTT) for bacterial inactivation under dual light irradiation at 660 and 808 nm; (**B**) In vivo assessment of the wound regions at Days 0, 2, 4, 8 and 14; (**C**) Spread plate assay for *S. aureus* obtained from the infected tissues on Day 4 (figure adapted from [85] with permission of Elsevier).

A hydrogel composite based on Ag_3_PO_4_ and MoS_2_ was also reported by Zhang and co-workers as an efficient light-assisted material for wound healing under co-irradiation with visible (660 nm) and near-infrared (NIR) (808 nm) lights (Figure 29) [85]. In this approach, the authors explored the high efficacy of Ag_3_PO_4_ in generating ROS with reduced toxicity due to its adsorption in MoS_2_, which minimized the waste of the toxic Ag(I). MoS_2_ nano-slices obtained by a hydrothermal growth approach were used to deposit the Ag_3_PO_4_ nanoparticles. The Ag_3_PO_4_/MoS_2_ hydrogel was obtained by dissolving these Ag_3_PO_4_/MoS_2_ composites in PVA followed by several freeze–thaw cycles. The photophysical and biological assessment showed that the Ag_3_PO_4_/MoS_2_ hydrogel was able to generate ROS with better efficacy under 660 nm light irradiation, while under 808 nm (NIR) light irradiation, more heat was produced. Taking advantage of this synergism between PDT and photothermal therapy (PTT), the hydrogel composite was able to kill almost all bacteria after dual light irradiation for 5 min at an irradiance of 0.6 W/cm^2^. Furthermore, the Ag_3_PO_4_/MoS_2_ hydrogel composite also showed an efficient promotion of the healing of wounds infected with *S. aureus*, when compared with a pure hydrogel and medical gauze (MG), without causing noticeable organ abnormalities in rats.

**Figure 30:**
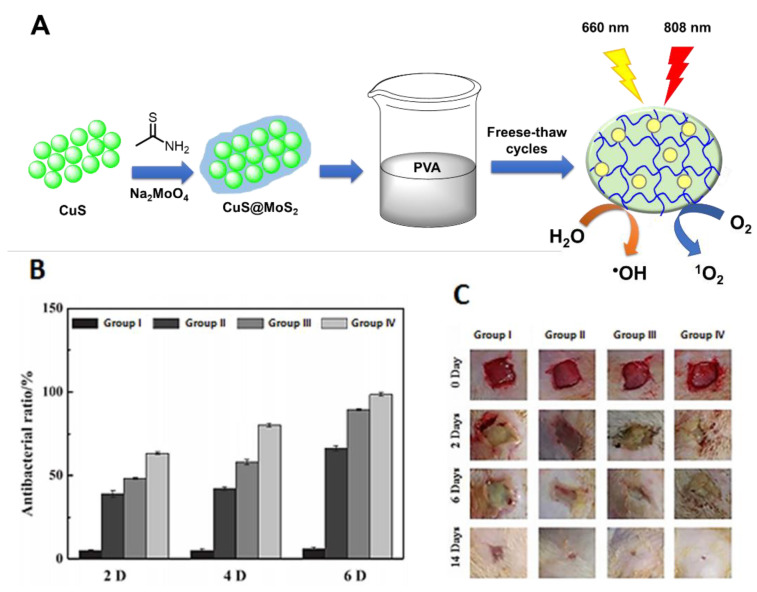
(**A**) Schematic of preparation of CuS@MoS_2_–poly(vinyl alcohol) (PVA) hydrogel and ROS production after dual irradiation with visible (660 nm) and near-infrared (NIR) (808 nm) light; (**B**) Antibacterial rates after 2, 4 and 6 days of therapy; (**C**) Photographs of *S. aureus*-induced wound infections at days 0, 2, 6 and 14 (figure adapted from [86] with permission of Elsevier).

In the same context of exploring the synergistic effects rendered by hyperthermia and ROS, Zhang and co-workers [86] reported the preparation of another promising photoactive hydrogel composite based on PVA but now embedded with CuS@MoS_2_ microspheres (Figure 30A). After 15 min of co-irradiation at 660 nm (visible light) and 808 nm (NIR light) with an irradiance of 0.2 mW/cm^2^, the hydrogel was able to inactivate 99.3% of *E. coli* and 99.5% of *S. aureus*. Three different experimental groups were compared in this study, including wounds treated with a (I) sterile wound dressing, (II) blank hydrogel, (III) CuS-incorporated hydrogel and (IV) CuS@MoS_2_-incorporated hydrogel composite (Figure 30B). Due to the synergistic effect between PDT and PTT, an improvement in the bacterial membrane permeability was considered to facilitate the bacterial destruction by ROS. Additionally, the improvement in wound healing (Figure 30C) by the CuS@MoS_2_-incorporated hydrogel was justified by the promotion of hypoxia-inducible factor-1 (HIF-1α) and vascular endothelial growth factor (VEGF) expression, and subsequent vascularization at the wound sites.

**Figure 31:**
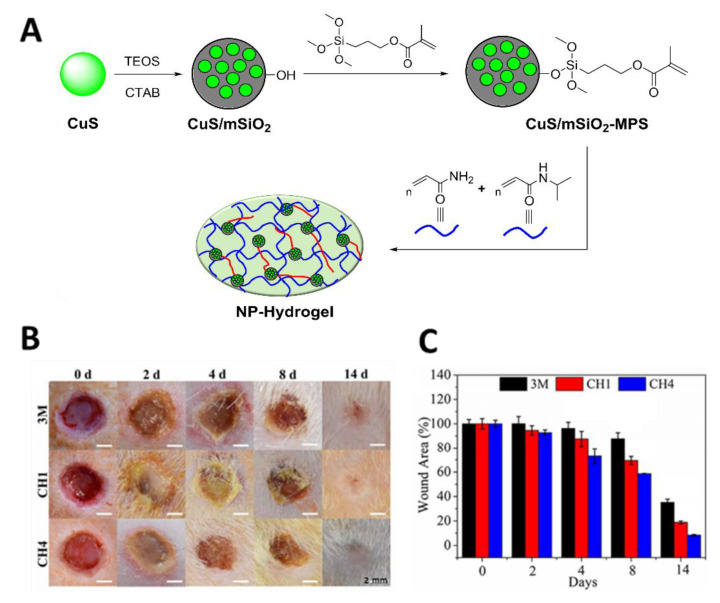
(**A**) Schematic illustration of the synthetic route for preparing a CuS/mSiO_2_ NP hydrogel), where TEOS = tetraethyl orthosilicate and CTAB = hexadecyltrimethylammonium bromide; (**B**) Photographs of *S. aureus*-infected wounds treated with different dressings at time points of 0, 2, 4, 8 and 14 days; (**C**) Wound area evaluation over the treatment duration (figure adapted from [87] with permission of Royal Society of Chemistry).

In 2018, Wu and co-workers [87] reported that an NPS hydrogel composed of 3-(trimethoxysilyl)propyl methacrylate (MPS, 97%) and mesoporous silica (mSiO_2_) modified with CuS@NPs (Figure 31A) possessed antibacterial efficacy against *S. aureus* (99.80%) and *E. coli* (99.94%) and was able to accelerate wound healing after irradiation with NIR light (808 nm) for 10 min at an irradiance of 2 W/cm^2^ (Figure 31B,C). Three different experimental groups were compared, including wounds treated with (I) a 3M wound dressing (commercial control); (II) CH1, a pure hydrogel; and (III) CH4, a hydrogel embedded with CuS NPs in 1.5 mg mL^-1^. The found behavior was justified considering the combined effects of hyperthermia, the formation of radical oxygen species, and the copper ion release produced during the CuS@NP NIR light irradiation. The controllable copper ion release induced by the hydrogel obtained from *N*-isopropylacrylamide (NIPAAm) and acrylamide (AAm) (Figure 33A) was considered important for stimulating fibroblast proliferation and angiogenesis, leading to both antibacterial effects and skin tissue regeneration.

**Figure 32:**
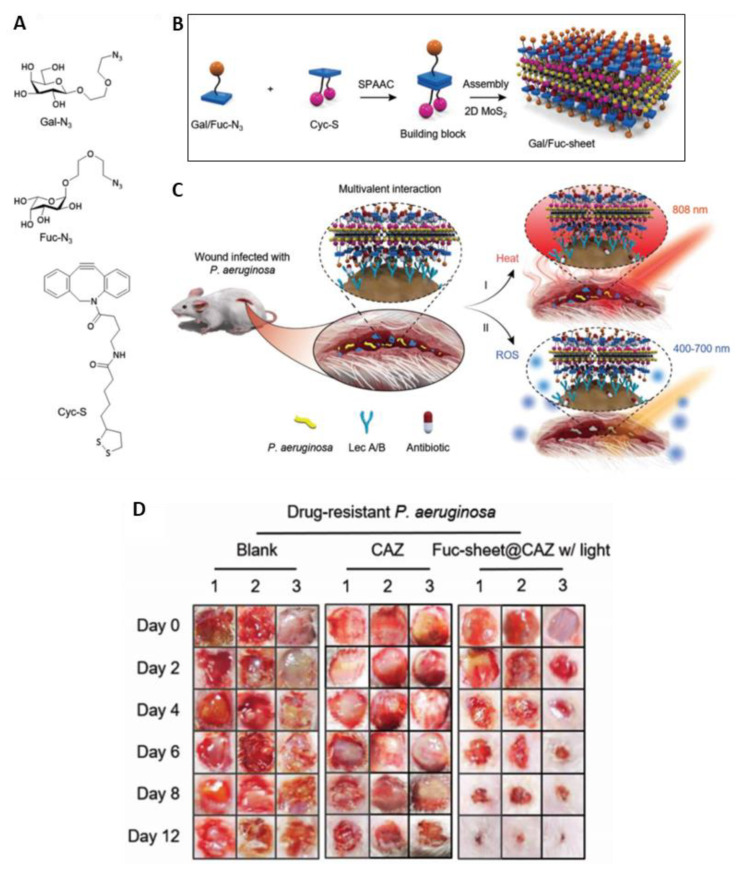
(**A**) Structure of azido-galactoside (Gal-N_3_), azido-fucoside (Fuc-N_3_), and α-lipoic acid-coupled cyclooctyne (Cyc-S); (**B**) Schematic illustration of glycosheet formation; (**C**) Double light-driven therapy of wound infected by *P. aeruginosa*; (**D**) Photographs of wounds treated in the presence of glycosheets for multidrug-resistant *P.* *aeruginosa* (figure adapted from [88] with permission of John Wiley and Sons).

In 2019, Hu and coworkers [88] reported the development of two multivalent glycosheets based on galactose and fucose, immobilized on the surface of thin-layer MoS_2_ (Figure 32A,B), and their efficacy against the multidrug-resistant *P. aeruginosa* in infected wounds under double light-driven therapy. The glycosyl building blocks were obtained from the strain-promoted alkyne copper-free click reaction using the adequate azido glycosides (Gal-N_3_ or Fuc-N_3_) and a cyclooctyne coupled to α-lipoic acid (Cyc-S), followed by their assembly into 2D MoS_2_ sheets. These glycosheets, under NIR (808 nm, 1 W/cm^2^) light irradiation, showed that the thermal effect can release antibiotics in situ and increase bacterial membrane permeability (Figure 32C). On the other hand, the white light-driven ROS production at an irradiance of 1 W/cm^2^, for 1 h, led to higher bacterium killing. The study showed that the targetability, together with the light sensibility, of the glycosheets enabled a highly effective and optically controlled therapeutic regime for the healing of wounds infected by multidrug-resistant (Figure 32D), as well as clinically isolated, *P. aeruginosa.*

**Figure 33:**
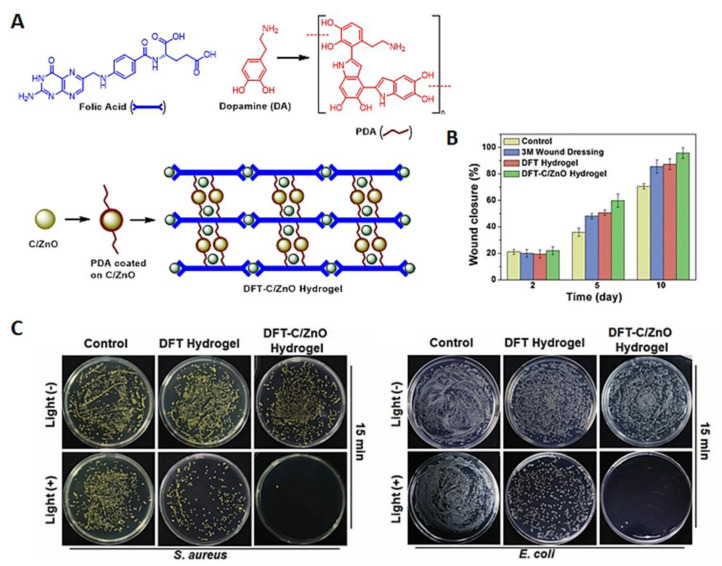
(**A**) Preparation of DFT-C/ZnO hydrogel, where PDA = polydopamine; (**B**) Wound area at different treatment time points; (**C**) Representative images of viable *S. aureus* and *E. coli* grown on different samples with or without 15 min dual-light irradiation (figure adapted from [89] with permission of John Wiley and Sons).

The injectable DFT-C/ZnO-based hydrogel (Figure 33A) was obtained by wrapping carbon quantum dots decorated with ZnO (C/ZnO), with an assembly obtained from dopamine (DA) and folic acid (FA) crosslinked by Zn^2+^ (DFT). The DFT-C/ZnO-based hydrogel also showed a great potential to accelerate wound closure (Figure 33B) and an effective antibacterial efficacy against *S. aureus* (99%) and *E. coli* (99.9%) under dual light irradiation at 660 and 808 nm for 15 min (Figure 33C). The hybrid hydrogel showed great potential for the in vitro reconstruction of bacterium-infected tissues, due to the promotion of fibroblast growth associated with the zinc ion release from DFT-C/ZnO-based hydrogels [89].

**Figure 34:**
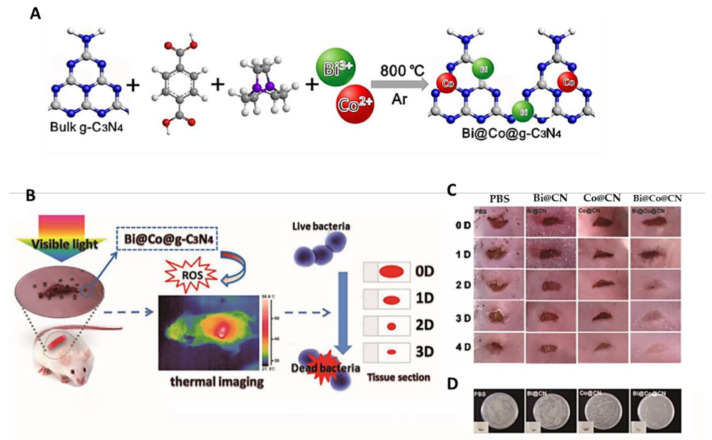
(**A**) Schematic illustration of Bi@Co@CN nanocomposite preparation; (**B**) The concept of a Bi@Co@CN photocatalyst as an efficient antimicrobial agent; (**C**) Photographs of mouse wound treated with PBS, Bi@CN, Co@CN and Bi@Co@CN; (**D**) Bacteria isolated from wound tissue cultured on agar plates (figure adapted from [90] with permission of Elsevier).

The composite Bi@Co@g-C_3_N_4_ (Figure 34A,B) based on graphitic carbon nitride (*g*-C_3_N_4_) and Bi/Co NP was reported as an effective PS for eradicating *S. aureus* (>99.999% within 20 min) and MRSA, due to its strong ability to generate ROS and thermal energy under simulation with visible light at an irradiance of 100 mW/cm^2^ for 30 min [90]. The composite was obtained by adding bulk *g*-C_3_N_4_ to a mixture of Bi(NO_3_)_3_ and Co(NO_3_)_2_ metal salts in DMF in presence of 1,4-benzenedicarboxylic acid and triethylenediamine, followed by thermal condensation at 800 °C.

The in vitro results revealed that the Bi@Co@g-C_3_N_4_ materials, under visible light irradiation, generate ROS and heat, which promote severe damage in bacterial cell membranes and affect the virulence factors produced by bacteria, leading to *S. aureus* death. Moreover, an in vivo assessment confirmed their efficiency in healing infected wounds (Figure 34C,D) without any detectable damage to major organs.

## 3. Final Remarks

This review is a comprehensive contribution concerning the performance of different non-porphyrinic PS classes for wound healing. Considering the importance of developing new PSs with appropriate structural, photophysical and photochemical features, for this emergent research topic, parameters such as the backbone PS structure, light source and wound target characteristics should be considered and deeply studied. However, the study of other factors such as the development and improvement of PS delivery conditions and the involvement of additional components in the photosensitizing system, to promote the PS effect enhancement and the wound healing process, has become a major issue for the scientific community in the last few years.

Since the late 1990s, PSs have been tested for skin incision photobonding; nevertheless, more recently, PSs’ usefulness for microorganism inactivation during wound healing has been considered, leading to a synergetic relationship with their photodynamic activity, preventing the development of microbial infections and allowing more effective wound healing. These findings led to an optimization of PDT conditions and parameters for different types of wound infections. In fact, a wide range of PSs discussed in this review have proved to be effective in reducing the bacterial load while improving the healing rate of skin wounds. However, the PS efficiency is strongly dependent on a high number of variables, such as PS type and concentration, wavelength irradiation, light dose, wound type and location, infected and non-infected wounds, and microorganism strain.

In the last few years, many authors have explored PS integration in several solid matrices, such as nanoparticles or hydrogels, to enhance the photodynamic process effectiveness in microbial inactivation and the wound healing process. These systems bring additional advantages of biocompatibility and regenerative conditions that were not possible to accomplish using non-supported PS. This efficiency is strongly related to the synergism between both the photoactive molecule and the solid matrix used as a support.

The development of polymeric hydrogels containing the PS molecule is really promising. The polymeric component is usually chemically suitable for being accepted by the surrounding environment of the skin tissues, thus helping the PS to play its role in the regenerative process. The tridimensional template, which is usually provided within the hydrogel, allows a high rate of the growth, division and migration of new cells, to optimize the formation of the new tissue favored by the action of the photosensitizing molecule. When a delivery template such as a hydrogel is used, it creates a water-donating vehicle for the wound site that helps to maintain a moist environment, which is crucial for the delivery of nutrients and immune defenses to the wound surface, allowing the acceleration of the regenerative process, acting as a “sidekick” of the photodynamic action.

Therefore, the development of new PSs with improved features, as well as their immobilization in a covalent or non-covalent fashion in biocompatible and stimulating materials to improve the synergism between both agents in wound healing, will continue to be a major goal, involving researchers from several scientific fields, such as organic chemistry, (nano)materials and medicine.

## Abbreviations

AAm	Acrylamide
aPDT	Antimicrobial photodynamic therapy
BP	Black phosphorus
CFTR	Cystic fibrosis transmembrane conductance regulator R
CLP	Collagen-like peptides
CMC	Carboxymethyl cellulose
CS	Chitosan hydrogel
DA	Dopamine
DFT	Directional freezing–thawing
ECM	Extracellular matrix
FA	Folic acid
FAK	Focal adhesion kinase
FDA	Food and Drug Administration
Fmoc-FF	*N*-fluorenylmethoxycarbonyl diphenylalanine
*g*-C_3_N_4_	Graphitic carbon nitride
GFs	Growth factors
HÁ	Hyaluronate
HIF-1α	Hypoxia inducible factor-1
HY	Hypericin
ICG	Indocyanine green
LED	Light-emitting diode
LLLT	Low-level laser therapy
MB	Methylene blue
min	Minute
MMPS	Metalloproteinases
m-PEG	Poly(ethylene glycol)monomethyl ether
MRSA	Methicillin-resistant *Staphylococcus aureus*
NF	Nanofiber
NIPAAM	*N*-isopropylacrylamide
NIR	Near-infrared
NP	Nanoparticle
PAAm	Poly(allylamine)
PB	Photobonding
PCL-PEG	Polycaprolactone-poly(ethylene glycol)
PDT	Photodynamic therapy
PEG-Ac	Polyethylene glycol-acrylate
PLL	Poly-*L*-lysine
PS	Photosensitizer
PTB	Photoactivated tissue bonding
PVA	Poly(vinyl alcohol)
PTT	Photothermal therapy
RB	Rose Bengal
ROS	Reactive oxygen species
SDBS	Sodium dodecylbenzenesulfonate
TBO	Toluidine O blue
TCP	Tissue culture plate
UPNP	Upconversion nanoparticles
VEGF	Vascular endothelial growth factor
